# Using intervention mapping to develop a home-based parental-supervised toothbrushing intervention for young children

**DOI:** 10.1186/s13012-016-0416-4

**Published:** 2016-05-06

**Authors:** K. A. Gray-Burrows, P. F. Day, Z. Marshman, E. Aliakbari, S. L. Prady, R. R. C. McEachan

**Affiliations:** 1 School of Dentistry, Clarendon Way, Leeds, LS2 9JT UK; 2 School of Clinical Dentistry, Claremont Crescent, Sheffield, S10 2TA UK; 3 Department of Health Sciences, University of York, York, YO10 5DD UK; 4 Bradford Institute for Health Research, Duckworth Lane, Bradford, BD9 6RJ UK

**Keywords:** Intervention mapping, Toothbrushing, Parents, Children

## Abstract

**Background:**

Dental caries in young children is a major public health problem impacting on the child and their family in terms of pain, infection and substantial financial burden on healthcare funders. In the UK, national guidance on the prevention of dental caries advises parents to supervise their child’s brushing with fluoride toothpaste until age 7. However, there is a dearth of evidence-based interventions to encourage this practice in parents. The current study used intervention mapping (IM) to develop a home-based parental-supervised toothbrushing intervention to reduce dental caries in young children.

**Methods:**

The intervention was developed using the six key stages of the IM protocol: (1) needs assessment, including a systematic review, qualitative interviews, and meetings with a multi-disciplinary intervention development group; (2) identification of outcomes and change objectives following identification of the barriers to parental-supervised toothbrushing (PSB), mapped alongside psychological determinants outlined in the Theoretical Domains Framework (TDF); (3) selection of methods and practical strategies; (4) production of a programme plan; (5) adoption and implementation and (6) Evaluation.

**Results:**

The comprehensive needs assessment highlighted key barriers to PSB, such as knowledge, skills, self-efficacy, routine setting and behaviour regulation and underlined the importance of individual, social and structural influences. Parenting skills (routine setting and the ability to manage the behaviour of a reluctant child) were emphasised as critical to the success of PSB. The multi-disciplinary intervention development group highlighted the need for both universal and targeted programmes, which could be implemented within current provision. Two intervention pathways were developed: a lower cost universal pathway utilising an existing national programme and an intensive targeted programme delivered via existing parenting programmes. A training manual was created to accompany each intervention to ensure knowledge and standardise implementation procedures.

**Conclusions:**

PSB is a complex behaviour and requires intervention across individual, social and structural levels. IM, although a time-consuming process, allowed us to capture this complexity and allowed us to develop two community-based intervention pathways covering both universal and targeted approaches, which can be integrated into current provision. Further research is needed to evaluate the acceptability and sustainability of these interventions.

## Background

Dental caries (tooth decay) is a worldwide public health problem, with millions of children experiencing caries in their primary teeth (the first set of teeth which erupt at approximately 6 months of age and exfoliate between the ages of 6 and 12 years old) [1, 2]. Moreover, there are marked health inequalities, such that those of lower socioeconomic status experience poorer oral health [3, 4]. In the UK, for example, an average of 31 % of 5-year-old children have obvious dental caries, with this figure increasing to 41 % in children from more deprived areas compared to 29 % living in more advantaged areas [5]. Caries is important to address as experience at this young stage is a key predictor of future oral health in adolescence and adulthood [6]. Dental caries in young children has significant impacts on health, social and intellectual development, including pain, eating difficulties, speech impairments, and significant morbidity to the child, and financial costs to the family and society [7–13].

Dental caries is preventable, and one key target behaviour of ensuring good oral health for children is through twice daily toothbrushing with fluoride toothpaste with supervision from a parent. UK guidelines recommend that from the age of primary tooth eruption (approximately 6 months old) up to the age of 7 years old, parents should supervise their child’s toothbrushing, known as parental-supervised toothbrushing (PSB) [11, 14, 15]. This is a dyadic process [16], which entails parents actively brushing their children’s teeth and children allowing their teeth to be brushed; as such, it is a complex behaviour with many influences at both individual (parent and child separately) and interpersonal levels (parent and child interactions). Furthermore, PSB is a complex behaviour as it is composed of a collection of behaviours beyond oral health practices, such as parenting; and due to the various socio-ecological influences on PSB, it can be a difficult behaviour to perform.

Evidence shows that PSB can lead to a 15 % reduction in dental caries and when begun before the age of one can double the chances of being free of obvious caries at preschool age [17]. This reduction in caries is most likely the result of the protection fluoride provides and more effective plaque removal by parents [18, 19]. However, little advice is provided on what PSB means and how to implement PSB, with few parents provided with advice on how to brush the teeth of their young children [16, 20, 21]. In addition, there is a lack of guidance for healthcare workers, dental teams and nursery nurses on how to support parents to implement PSB into their child’s daily lives [11, 14, 15]. Therefore, it is unsurprising that, 50 % of 5-year-olds in the UK brush their teeth without supervision [18], with observational studies reporting substantial inadequacies in the efficacy and frequency of brushing [22], leading to a significantly greater risk of developing dental caries [17, 23, 24].

Due to the rising concern of the problems caused by dental caries in young children and the impact of this on later life, there has been a drive to produce interventions to improve oral health in children. These have been particularly focused on school-based toothbrushing programmes [13]. Evidence suggests that such interventions can be effective in nursery and school settings [25, 26]. However, there are problems with school-based interventions. First, they only target children of a later age; thus, dental caries may already be a significant problem before the intervention is available. Second, the interventions place extra burden on school staff. Third, it has been suggested that these effects are not necessarily maintained [27] and it is unclear whether school-based brushing has an impact on home-based toothbrushing [25]. Indeed, there is evidence to suggest that school-based toothbrushing programmes can have a detrimental effect on home-based brushing as some parents perceive responsibility for brushing transferring to the role of the school [25]. Nevertheless, such interventions have been shown to significantly reduce caries in permanent teeth in high-risk children recruited at 5 years old to a 2-year intervention and may be one of the best ways of overcoming the barrier of cost of dental resources for low-income families [28].

Home-based interventions have the advantage of targeting PSB at an early age and habitualising important oral health behaviours, but compared to school-based interventions, programmes involving parents have been investigated to a lesser extent. A recent review of 18 interventions concluded that the majority were poorly described, lacked a sound theoretical grounding, and effects were mixed, with only 8/18 yielding any significant results [29]. MRC guidance for the development of complex interventions recommends the use of theory to ensure that interventions target factors which are likely to have an impact on the desired outcome and involvement of stakeholders to ensure that interventions developed are feasible and acceptable [30]. Within the review discussed previously, only five studies based interventions on a theoretical framework; for example, Freudenthal and Bowen [31] and Weber-Gasparoni et al.’s [32] interventions were based on motivational theories, including the Transtheoretical Model [33] and self-determination theory [34], respectively, targeting behaviour change at the level of the individual. However, as research has shown PSB is an interpersonal behaviour and influenced by a range of factors not only beyond the individual but also beyond motivation, thus, a more comprehensive approach is needed to target the barriers to PSB at all levels of influence (individual, interpersonal and environmental). Within the UK, national guidance states that future interventions need to use the wider workforce in the community to deliver interventions to ensure their sustainability and implementation [13, 35, 36] and to develop interventions which address the health inequalities that persist in oral health [3, 4]. In order to achieve these aims, it is necessary to include key stakeholders including commissioners, health practitioners and community members in the development of new interventions. There is thus a clear need for the development of interventions to promote PSB, which take into account theory, evidence and context in their development.

It is vital to develop appropriate evidence-based interventions to encourage adoption of PSB, which are based on sound evidence, and can be integrated within existing delivery channels. Intervention mapping [37] is a protocol for the development of complex interventions, which encompasses recommendations of the MRC guidance in its approach. Specifically, it (i) takes into account theory and evidence detailing how change is likely to occur, (ii) takes an ecological perspective to the development process and explicitly addresses individual, interpersonal, organisational, community and societal influences on behaviours and outcomes and (iii) is grounded in community participation allowing contributions from a range of stakeholders to contribute to the development process. Thus, it was deemed particularly suitable for development of an intervention to address a complex behaviour such as PSB with influences from multiple levels. Although it has been used in a wide variety of other contexts including increasing physical activity [38] and preventing childhood obesity [39], it has never been applied to oral health behaviours, despite being recommended as a means of developing high-quality interventions to improve oral health outcomes [40].

The aims of this paper were to describe how the IM approach was used to develop a home-based parental-supervised toothbrushing intervention to reduce dental caries in young children and to explore the strengths and limitations of this approach. We used the IM approach to develop an intervention which would be complementary to existing service provision and improve provisions, based on behaviour change theory, and designed to target deprived communities in most need.

## Methods

The IM process comprises of six steps: (1) needs assessment; (2) identification of outcomes and change objectives; (3) selection of methods and practical strategies; (4) production of a programme plan; (5) adoption and implementation and (6) development of an evaluation plan.

### Step 1: needs assessment

#### Intervention development group

In order to guide the process, a multi-disciplinary intervention development group was convened. The intervention development group (*n* = 19) included parents, dental practitioners, community workers, local councillors, healthcare practitioners and academics. The group was led by a behavioural scientist and met once a month over a 4-month period to discuss oral health; barriers and facilitators to PSB, including findings from the systematic review and qualitative interviews (see below); and intervention development (i.e. outcomes, delivery, practical strategies and feasibility). Contact was additionally made with organisations that could inform intervention design and delivery (see Table 1 for the composition of the intervention development group).

**Table 1:** Composition of intervention development group

Role	Number
^a^Parents	2
^a^Central Eastern European community worker	1
^a^Oral health promotion staff	2
Public health England	1
^a^Experts in public health	3
^a^Health visitor	1
^a^School nurse	1
^a^Dental practitioners	4
^a^Healthcare interpreter	1
^a^Parent co-ordinator	1
Early years/children’s centres	4
^a^Expert on inequalities in health	1
^a^Experts in behaviour change	2
Experts in education and parenting programmes	2
Children’s charities	1

^a^Members of intervention development group

#### Systematic research review

A systematic review [29] was undertaken to identify all the relevant literature on the prevalence of PSB, barriers and facilitators to PSB and any home-based parent-driven toothbrushing interventions in children up to the age of 7. Database searches identified 3221 papers of which 95 papers were included. In order to build the limited existing application of theory in this area, we mapped the barriers and facilitators of PSB identified in the studies, as well as the barriers addressed in intervention studies onto the Theoretical Domains Framework (TDF) [41]. The TDF is a comprehensive list of 12 theoretical determinants of behaviour derived from 33 behaviour change theories and has been successfully used to identify important theoretical determinants of behaviour in a wide array of contexts.

#### Qualitative interviews

Qualitative semi-structured interviews were conducted with parents/carers of children under 7 years (*n* = 27). Children under 7 were interviewed as “children need to be helped or supervised by an adult when brushing until at least seven years of age” [15, 36]. Participants were purposively sampled to ensure that parents living in deprived areas of Bradford and Barnsley (UK cities with significant levels of deprivation and ethnic diversity) ranged in gender, children’s age, ethnicity, native language, dental caries experience and dental attendance patterns. Potential participants were identified from previous research projects, children’s centres and community dental clinics. Data saturation was reached after 27 interviews. Thirteen interviews took place at participants’ homes, 11 were conducted at a children’s centre, 2 were conducted at a research institute and 1 interview was conducted by telephone. Participants were 22 mothers, 2 fathers and 3 grandmothers. The aims of these interviews were to explore the oral health behaviours of parents of young children and to identify the theoretical barriers and facilitators to PSB. The interviews were based on the Theoretical Domains Framework and analysed using framework analysis [41]. See Table 2 for examples of how barriers were mapped onto the different theoretical domains.

**Table 2:** Derivation of Theoretical Domains Framework from needs assessment

Theoretical domains from TDF	Number of times domain identified as a barrier/facilitator in the systematic review**	Qualitative interviews—example quotes
Knowledge	43	“I don’t think they’ve ever told us that under the age of 7 you should brush your kids teeth”
Skills	17	“I have to say to her give me a turn and then it’s your turn to brush her teeth and she has her turn…”
Social/professional role and identity	3	“It is my responsibility because they’re my kids, I brought them into this world so it’s my job to give them the best upbringing”
Beliefs about capabilities	13	“…all the time I am worrying…like if I’m doing it right…”
Beliefs about consequences	21	“you can actually smell their breath like when their talking to you and if they’ve not brushed their teeth it really really smells”
Motivation and goals	13	“I’d have think its lacking motivation more than anything – obviously I do want them clean but I think with me what it is its just sort of finding the hours in the day to get round and do everything and a lot of the time were just so busy doing everything it’s sort of quickly in and quickly out
Memory, attention and decision processes	0	“I just think I forget cause I’ve only so many hours in the day to do things”
Environmental context and resources	22	“…but at night because she’s sort of in and out doing things she does tend to forget she’s got to come in and do them, and when I go up to bed cause I go up to bed with her, I will say to her bathroom first and teeth done and that’s when you start with your problems! She just doesn’t want to do them at night”
Social influences	10	“You see her Dads a problem as well – he doesn’t do his as regular, now her Granddad does, he’s always in the bathroom and he’s always reminding her, he’s brilliant doing his”
Emotion	5	“I’m really happy about it; I prefer brushing their teeth than asking them to do it, because when I do it I know it’s done properly”
Behaviour regulation	13	“…if I try to brush it for him he’ll throw a tantrum, he throws the toothbrush at me, toothpaste at me and just lay on the floor and start kicking his legs…”
Nature of behaviours	3	“but if parents encourage the kids every day or tell them or like me become a habit then it’s much more easier for them just getting used to it like a daily routine so they have to do it, they have to do it that’s it”

Findings taken from Aliakbari et al. [29] and Marshman et al. [21]

**n = 95 (Note: Studies were not mutually exclusive and could identify multiple domains)

### Step 2: identification of outcomes, performance objectives and change objectives

The next step in the process involved the detailed specification of the desired outcomes for the intervention. The overarching outcome of the current intervention was to reduce dental caries in young children. The goal of PSB is primarily to prevent caries in young children and secondarily to prevent existing caries from getting worse. All children are at risk for dental caries, and although some children are at higher risk, for example those with high levels of sugar in their diet, PSB is a behaviour that can be applied universally to support dental health. Although not irrelevant, caries status is therefore not a highly important factor in relation to the PSB behaviour. It was aimed to achieve this outcome by improving oral health through the promotion of oral health-related behaviours, primarily encouraging PSB. However, it was recognised that there are numerous influences on PSB (e.g. individual, interpersonal, organisational/community and environmental); thus, specific intervention outcomes were defined for each level of influence in line with the socio-ecological model [42] and scrutinised by the research team and intervention development group.

Following the specification of outcomes, performance objectives for each of the specified outcomes were defined. Performance objectives are a means of identifying the precise behaviours that must occur to achieve the specified outcomes. The final stage in this process required that the objectives of the intervention were stated in terms of the actual changes that need to occur in the theoretical determinants of behaviour. This is vital as it allows the intervention developer to identify the exact psychological constructs that need to change to have an effect on the performance objective and the programme outcome as a whole. Each performance objective was scrutinised by behavioural scientists (KG-B, RM) to identify the specific psychological determinants of behaviour useful in changing each performance objective. This was achieved by reflecting on the barriers faced by individuals to behavioural performance, which were mapped using the TDF. For example, if a performance objective was for parents to know what PSB means and how to perform PSB, appropriate theoretical determinants would be knowledge, skills and beliefs about capabilities (self-efficacy). This process is useful as it encourages intervention developers to precisely state what needs to be targeted to affect the performance objective and select appropriate evidence-based behaviour change techniques to address the contributing psychological constructs identified. This process resulted in a matrix specifying the performance objectives, the theoretical determinant of that behaviour and change objectives.

### Step 3: selecting methods and practical strategies

The next stage of the IM process was to identify theoretical methods deemed to be effective in changing theoretical determinants. Theoretical methods/behaviour change techniques [37, 43] were mapped against each determinant area by two behavioural scientists (KG-B, RM). Change objectives grouped under each determinant area were then operationalised into practical strategies. Practical intervention strategies were developed by the research team including behaviour change experts, with practical strategies identified in the systematic review [29], interviews [21], and by the intervention development group. In addition, practical strategies suggested by the team were shared with the intervention development group to gauge the feasibility of these strategies.

### Step 4: creating an organised programme plan

In the next step, an organised programme plan was created. This entailed outlining the scope and sequence of the intervention components, materials and protocols. The intervention development group and contact with wider organisations provided guidance as to the scope and implementation of the intervention. A large range of change objectives and potential strategies were identified at individual, commissioner and practitioner levels. The change objectives and practical strategies were filtered down to those that were feasible to target in the planned intervention (e.g. at practitioner and individual level).

### Step 5: creating an adoption and implementation plan

In the penultimate step, an adoption and implementation plan was formulated in consultation with the intervention development group. Consultations with key stakeholders related to health visiting services and parenting programmes discussed how the interventions would be integrated within their existing services, as well as how and when they would be delivered and by whom. Training guides and lesson plans were mapped out addressing key barriers to PSB for health visitors and parenting programme facilitators.

### Step 6: creating an evaluation plan

The last step of IM is to create an evaluation plan. This step was not in the scope of the current paper and will be reported elsewhere.

## Results

### Step 1: needs assessment

#### Identification of key barriers and facilitators

**Fig. 1:**
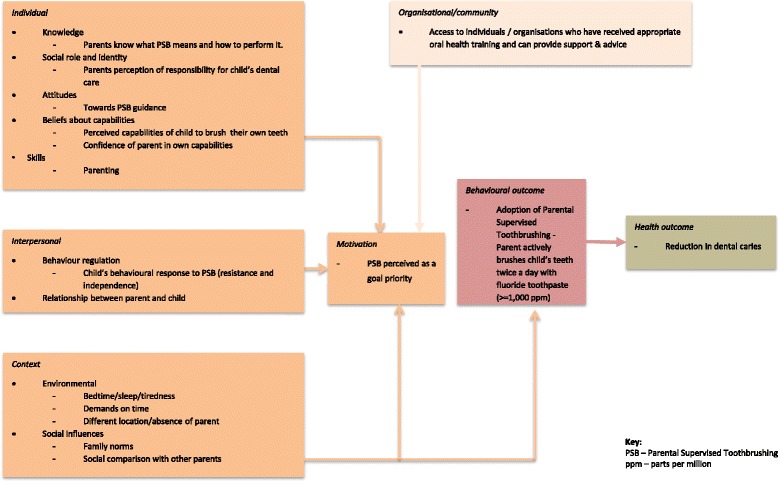
Logic model for parental-supervised toothbrushing with focus on parenting programme-based intervention

The systematic review conducted as part of this programme of work aimed to identify theoretical determinants useful in predicting and explaining PSB. Aliakbari et al. [29] found wide variation in the prevalence of PSB. The literature suggests that there are a range of barriers to PSB, which were mapped onto the TDF. This mapping process revealed that the main barriers fell into the theoretical categories of knowledge, beliefs about capabilities (self-efficacy), beliefs about consequences (attitudes), behaviour regulation, social influences, environmental context and resources, emotion and the nature of the behaviours (See Table 2 for further detail on the frequency of these barriers). For example, one key barrier is parental self-efficacy. Many parents feel they lack the knowledge, skills and confidence to appropriately brush their child’s teeth. Furthermore, in a number of studies, many parents expressed that although they try to implement good oral hygiene practices, they are faced with a difficult situation where the child does not want to have their teeth brushed by a parent, therefore leaving parents reticent to continue with toothbrushing routines. These barriers were further borne out with the findings from Marshman et al. [21], with the interviews revealing that parents were knowledgeable about the importance of brushing twice-a-day with fluoride toothpaste and the consequences of developing dental caries. However, parents’ interpretation of what ‘supervised brushing’ means was less clear and in contradiction to the clinical guidelines meaning. For instance, whilst parents brushed the teeth of their children under 1 year of age, this was not continued up to the recommended 7 years of age with difficulties reported by parents gaining the child’s co-operation. Facilitators to PSB tended to be the reversal of these barriers; for example, if a barrier was a lack of knowledge about PSB guidelines and how to brush their children’s teeth, a facilitator was improved knowledge of what PSB means and how to perform PSB. However, other facilitators included the use of rewards, such as sticker charts for children. Using the TDF as a guide, we summarised the theoretical determinants of PSB behaviours identified via our systematic review and qualitative research into a logical model (see Fig. 1).

#### Intervention development group

Discussions with the intervention development group and wider organisations highlighted the importance of parenting skills in the performance of toothbrushing behaviour, particularly routine setting and behaviour management. In addition, it was suggested that there is a need to consider the wider family and culture and how family background and experience can influence parents handling of their child’s oral health. Furthermore, the intervention development group and wider organisations expressed that any intervention targeting PSB would benefit from a non-dental user-friendly setting that is interactive, including various visual demonstrations and materials.

A number of key findings emerged from the needs assessment that are summarised in Table 3. The needs assessment led to the essential development of a logic model identifying key areas to target and our strategic short and long-term goals (Fig. 1). This process also identified two potential vehicles of delivery for the intervention: health visitors and parenting programmes.

**Table 3:** Key lessons learnt from needs assessment

Key lessons learnt about barriers/facilitators and intervention content and delivery
• Need to educate parents as lack of parent-targeted oral health programmes currently. • Education on oral health needs to begin early in a child life. • Family background, including parents own oral health, and the influence of the extended family and culture can have an impact on oral health. • Need to emphasise the personal responsibility of parents to take care of child’s oral health. • Need to highlight dental caries preventable and show consequences of brushing vs. not brushing. • Oral health messages need to be consistent. • Intervention needs to be user-friendly, fun and interactive (e.g. peer support, use of videos, practical demonstrations, phone Apps and novelty toothbrushes). • Wider parenting skills (e.g., routine setting and behaviour management) highly important to toothbrushing behaviour. • Language barriers and cultural sensitivity are key considerations in the development of an intervention. • Signposting to existing services would be useful to parents. • Interventions should be delivered through existing community services (e.g. health visitors, children’s centres). • Parenting programmes are a potential means of addressing wider parenting skills and delivering an intervention with an existing community provision.

The Healthy Child Programme is the government’s early intervention and prevention public health programme delivered by heath visitors from 0 to 19 years of age. One of the aims of this programme is to raise awareness about children’s oral health during the home developmental review sessions from 0 to 5 years of age [35]. This was therefore seen as a universal means of delivering a PSB intervention. However, it was acknowledged by the group that due to capacity limitations, such an intervention would be unable to address all the barriers to PSB, and indeed, barriers relating to parenting skills would benefit from a targeted approach. Thus, parenting programmes were suggested as a vehicle of intervention delivery for a targeted programme. Parenting programmes (e.g. Henry, Incredible Years and Family Links) are increasingly being commissioned and delivered in the community and broadly aim to improve the health, social and emotional wellbeing of parents and children by improving parenting skills. Therefore, this existing context was seen as a positive means of promoting both parenting and oral health skills. It resultantly became clear that there were two possible intervention pathways through the two different delivery vehicles: a universal approach (health visitor delivered) and a targeted approach (parenting programme delivered).

### Step 2: identification of outcomes, performance objectives and change objectives

The intervention development group agreed the overall outcome of the current intervention should be to reduce caries in young children. Using the socio-ecological model [42], outcomes were specified at individual, interpersonal, organisational/community and environmental levels (see Table 4 for the specific outcomes).

**Table 4:** Specific intervention outcomes by socio-ecological level

Overall outcome	Outcome level	Specific outcomes
Reduce dental caries in young children	Individual	Parent brushes child’s teeth, covering each tooth, twice a day with fluoride toothpaste up until 7 years of age.
	Interpersonal	Parent and child co-operate to perform PSB twice a day using fluoride toothpaste up until 7 years of age.
		Family members and friends who may take care of child to perform PSB when necessary and apply to child’s siblings and own family.
	Organisational/community	Appropriately trained individual within the community ensures families know what PSB is and how to perform PSB.
		Parents encourage and support other parents in the community with issues surrounding PSB.
	Environment	Home environment created that facilitates parent to brush child’s teeth twice a day with fluoride toothpaste up until 7 years of age.

The next stage of the process was to stipulate the performance objectives for each of the specific programme outcomes. This process was informed by theoretical knowledge about the determinants of behaviour as specified in the TDF. This list was then scrutinised and validated by the research team. Twenty-nine performance objectives and 117 accompanying change objectives were identified. These objectives were examined by the research team to assess which objectives required prioritising. Selected examples of change objectives for a sample of performance objectives can be seen in Table 5.

**Table 5:** Example of change objectives for selected performance objectives

Performance objective	Determinant	Change objective(s)
Parent actively brushes child’s teeth with fluoride toothpaste twice a day	Motivation and goals	Intend to brush child’s teeth with fluoride toothpaste twice a day
	Knowledge	Know how to brush child’s teeth
	Beliefs about capabilities (self-efficacy)	Express confidence in ability to actively brush child’s teeth with fluoride toothpaste twice a day
		Demonstrate ability to actively brush child’s teeth with fluoride toothpaste twice a day
	Skills	Develop skills to actively brush child’s teeth with fluoride toothpaste twice a day
		Demonstrate appropriate PSB (i.e. twice a day)
	Beliefs about consequences (attitude)	Increase recognition of importance of parent actively brushing child’s teeth with fluoride toothpaste twice a day
Parent perceives themselves as responsible for keeping their child’s teeth clean	Social role and identity	Perceive and take responsibility for brushing child’s teeth
	Beliefs about consequences (attitude)	Believe that being responsible for child’s toothbrushing will improve outcomes for child
	Social influence	Believe that others important to them think they should be responsible for brushing child’s teeth
Parent manages competing demands on time/resources	Knowledge	Know how to manage social demands on your time and resources (e.g. siblings, family problems)
		Know how to manage environmental demands on your time and resources (e.g. work commitments, financial issues)
	Beliefs about consequences (attitude)	Increase belief that proactive management of competing demands on time/resources will be beneficial
	Beliefs about capabilities (self-efficacy)	Express confidence in managing competing demands on time/resources
	Skills	Develop ability to manage competing demands on time/resources
		Demonstrate ability to manage competing demands on time/resources
	Social influences	Manage social and family pressures on time/resources
	Environmental contexts and resources	Manage environmental demands on time/resources
Parent copes with problems faced with PSB	Knowledge	Knows about potential problems and how to cope with them
	Skills	Develop coping strategies to manage problems faced with PSB
	Behaviour regulation	Identify strategies to manage child’s behaviour in response to PSB
	Beliefs about capabilities (self-efficacy)	Express confidence in ability to cope with problems faced with PSB
		Demonstrate ability to cope with problems faced with PSB
	Environmental context and resources	Identify environmental contexts that could lead to problems with performing PSB (e.g. tiredness)
		Develop strategies to overcome environmental problems (e.g., tiredness)
If in different location, parents to pack necessary equipment to perform PSB in new location	Environmental context and resources	Identify situations where child will be in location different to usual location PSB takes place
		Execute normal PSB routine in new location
	Knowledge	Know to continue brushing child’s teeth routine in new location
		Know to pack child’s toothbrush and toothpaste
	Beliefs about consequences (attitude)	Increase recognition of the importance to brush child’s teeth irrespective of location
	Beliefs about capabilities (self-efficacy)	Express confidence in ability to brush child’s teeth in new locations
		Demonstrate ability to brush child’s teeth in new locations
	Nature of behaviour (routine)	Organise PSB routine to take place in new location
		Execute PSB routine in new location

### Step 3: selecting methods and practical strategies

Examples of theoretical methods and practical strategies related to motivation and goals change objectives can be found in Table 6. For example, the change objectives in this area relate to increasing motivation to perform PSB and persist in the face of barriers that may emerge. Theoretical methods deemed potentially useful were goals and planning, prompts/cues and information on consequences. Considering these theoretical determinants, it was decided that practical strategies, such as using workbooks to allow parents to specify a series of implementation intentions regarding how, when and where they will perform PSB and encouraging the use of reminders and environmental cues to prompt PSB may be useful. Furthermore, the use of group discussion, workbook activities, video vignettes and leaflets can be used to convey the costs and benefits of engaging in PSB vs. not engaging in PSB.

**Table 6:** Examples of strategies for motivation and goals change objectives

Change objective	Theoretical method(s)	Practical strategy
Motivation and goals	Goals and planning	*Universal programme*
*Individual/interpersonal level*	Prompts/cues	Leaflet provided detailing UK PSB guidance and highlighting the pros/cons of parental involvement in toothbrushing a child’s teeth vs. not brushing
Intend to purchase appropriate fluoride toothpaste	Information on consequences	
Intend to brush child’s teeth with fluoride toothpaste twice a day		*Targeted programme*
Increase motivation to prioritise brushing child’s teeth		Ask parents to make a series of implementation intentions using a workbook
Increase motivation to allow parent to brush teeth		Intentions to purchase toothpaste and toothbrush When, where and how they will brush their child’s teeth Intentions to persist in the face of barriers Intentions to inform significant others of child’s toothbrushing routine
Increase motivation that persisting with brushing child’s teeth when faced with un-cooperative behaviour is a goal worth effort		
Increase motivation to persist with brushing child’s teeth in face of problems with PSB environment (i.e. tiredness)		
Intend to provide toothbrush and toothpaste to guardians looking after child		Encourage the use of reminders such as setting alarms for toothbrushing or using environmental cues (e.g. bath time)
Parent increases guardians motivation to brush child’s teeth in their absence		Information on the benefits and costs of parental involvement in brushing child’s teeth vs. not brushing child’s teeth provided in leaflet/video and/or group session with parents by a facilitator
Intend to brush child’s teeth at specific times and places twice a day, every day		Information accessible via University-hosted website

### Step 4: creating an organised programme plan

**Fig. 2:**
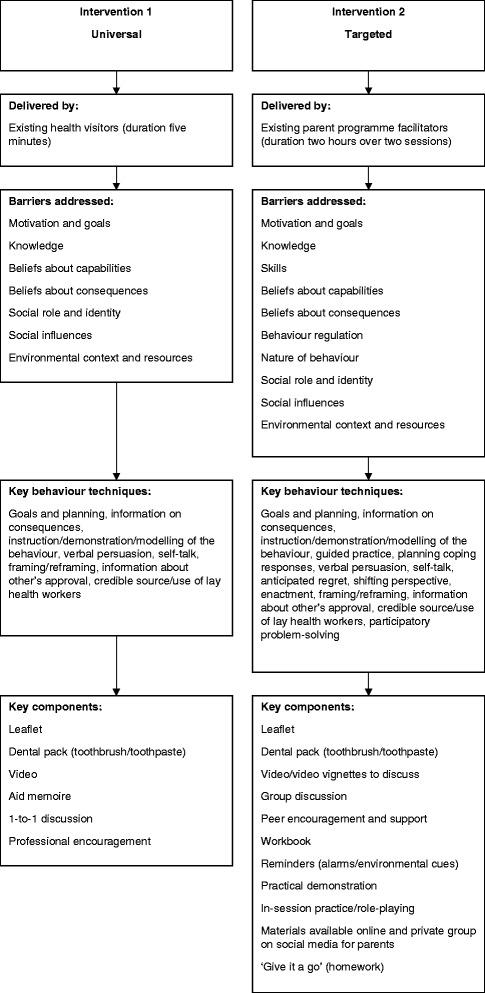
Diagram of the two intervention pathways, outlining the delivery, barriers addressed and key components of each intervention

The next stage required deciding on the scope and limitations of the intervention, translating the practical strategies into programme components and identifying methods of delivery that would be feasible and able to be implemented within existing provisions. A key barrier identified by the intervention development group related to effective parenting skills (particularly managing child behaviour), thus highlighting that a wider range of skills are needed beyond basic oral hygiene to effectively undertake PSB, skills that are not addressed by current oral health promotion activities. Parenting programmes offer the opportunity to provide more focused and intensive intervention, but they can be time-consuming and costly and thus not available as a universal intervention. Therefore, two intervention pathways were developed (Fig. 2). The first consisted of augmenting standard health visitor practice with additional materials targeting key barriers and the provision of further training to existing health visiting teams who currently deliver the universal Healthy Child Programme [35] to enable them to effectively intervene. This enabled the programme to be delivered universally to parents of all children. The second pathway included a more intensive targeted programme focused on building skills, particularly those relating to wider parenting skills, such as routine setting and behaviour management. The delivery of this intervention programme would be through parenting programmes. Targeted sessions were needed that could be either ‘woven into’ or ‘bolted onto’ existing parenting programmes and would focus on PSB.

The aim of the first intervention pathway would be to provide health visitors with enhanced oral health training and supportive materials (an aid memoire) to ensure standardised provision of oral health advice, with a focus on PSB. For parents, the intervention emphasis is placed on developing their knowledge, skills, positive attitudes and confidence specifically relating to child oral health and PSB. In addition, parents will be provided with a dental pack including a toothbrush, toothpaste and leaflet, as well as being directed to web-based video clips to further support their skill development. Such additional material is vital to facilitate further learning and skills development beyond the session as health visitors operate within strict time limits with a wide range of other developmental and parenting issues to be covered. Thus, due to capacity limitations, this intervention would address a proportion of the barriers to PSB. For example, parenting skills have been emphasised as key to the performance of PSB, but due to time restrictions, such a barrier could only be briefly discussed.

The second intervention pathway aims to address wider parenting skills as well as oral hygiene skills by delivering sessions that are embedded within existing parenting programmes. Currently, parenting programmes cover numerous parenting skills, including routine setting and child behaviour management. The sessions will target all the barriers identified to PSB, with particular emphasis on the barriers that emerge at three key time points in a child’s life between the age of 0 and 7 (tooth eruption (~6 months), 2 years and preschool age). These barriers being routine initiation, knowledge and skill development and behaviour management. Sessions would be led by a parenting programme facilitator; however, the session would be interactive with group discussion where parents identify their own barriers to PSB and identify strategies to overcome these barriers being a key component. These discussions would be further supported via practical demonstrations and in session practice of toothbrushing, group exercises based within a workbook and the display of video vignettes to stimulate discussion and problem-solving. In addition, parents would also receive a dental pack (toothbrush, toothpaste and leaflet) and have access to a website that would serve as a home for all the material delivered within the sessions that parents can access in their own time to further consolidate their skill development. Indeed, parents would be actively encouraged to practise what they have learnt at home and spread their learning to other family members and the wider community, with the second session allowing parents to report back on their progress and discuss difficulties encountered. Owing to the supportive nature of parenting programmes, parents with their peers can be guided to find a method which works for their children whilst ensuring appropriate oral health practices are adopted.

Training manuals have been created to accompany each intervention to ensure knowledge and standardise implementation procedures. The training for both interventions addresses the barriers to PSB of motivations and goals, knowledge, beliefs about consequences and capabilities, social influences, including social role and identity, and environmental context and resources. However, the targeted intervention training expands on this by also addressing the barriers of skills, behaviour regulation and the nature of the behaviour.

### Step 5: creating an adoption and implementation plan

As previously mentioned, it has been recommended that future oral health promotion interventions should aim to utilise the existing childhood workforce to provide a community-based intervention [13, 35, 36]. Therefore, with regard to the universal intervention, it was decided to deliver the intervention through health visitors that already have regular contact with parents and cover oral health albeit briefly within their existing provisions. In respect to the targeted programme delivered through parenting programmes, it was decided that the parenting programme facilitators would be best placed to deliver the intervention. Using existing facilitators confers a number of benefits, as facilitators: (1) are familiar with delivering an evidence-based programme, (2) are familiar with the local community and able to build a rapport with parents and (3) the intervention would be implemented within an existing community service, therefore increasing sustainability. The next stage of this process will be to define the clinical and process outcome measures for the intervention and to evaluate the feasibility of the two intervention pathways.

## Discussion

This paper aimed to describe in detail the process of using an IM approach to developing a home-based parental-supervised toothbrushing intervention to reduce dental caries in young children and to explore the strengths and limitations of this approach. This oral health intervention, to our knowledge, is the first to use an IM approach that incorporates evidence based on review, interview and stakeholder data, behaviour change theory, and has been co-developed with community and stakeholder input. Although the intervention has been developed within a UK context, the lessons learnt from using the IM process have relevance for researchers and practitioners internationally, especially considering the current paucity of evidence-based interventions and their failure to address all barriers to PSB.

The intervention process has identified the multiple barriers to PSB adoption by parents. The two proposed interventions (universal and targeted) provide differing levels of support. This design acknowledges that for some parents, the more targeted help is required for them to adopt PSB. Further work will be needed to identify which groups of parents need the different approaches and the efficacy of these interventions. We identified two key pathways which fit in with existing provision within the UK of health services to children and families: first, a universal intervention that enables key health professionals (i.e. health visitors) to help parents overcome key barriers to performing appropriate parental-supervised brushing in line with clinical guidelines. This involves the provision of training for health visitors and the provision of materials to parents. This lower cost intervention can be implemented within the current Healthy Child Programme pathway and thus has potential for high reach and scalability. Second, in recognition of the wider context in which toothbrushing takes place and the key role of parenting skills, a targeted programme embedded within parenting programmes run within existing community settings has been developed. These sessions not only address improving knowledge and skills related to PSB, but also wider parenting skills by utilising the teachings of the parenting programmes.

Both intervention pathways address key barriers to PSB among parents of young children using theoretically underpinned behaviour change techniques, though the universal intervention is limited in the number of barriers it addresses compared to the targeted intervention. The targeted programme has the advantage of addressing wider parenting skills, and this is vitally important as increasingly research is showing parenting skills to be fundamental to oral health practices.

Through our own needs assessment, our intervention development group expressed the invaluable nature of good parenting skills, and indeed, the systematic review we undertook identified parenting skills as a prominent determinant of toothbrushing practices [29]. Furthermore, recent qualitative research on PSB with parents has emphasised the fundamentality of parenting skills [16, 21]. The main barrier related to managing the behaviour of the child, as children displayed resistance to letting their parent carry out toothbrushing. Therefore, to ensure children’s teeth are cleaned sufficiently, parents must learn behaviour management skills to negotiate what can be a difficult encounter, which is a key component of all parenting programmes. Moreover, these issues highlight the importance of framing our specific outcomes for our interventions using the socio-ecological model, as to effect behaviour change, we clearly must look beyond the individual. PSB is an interpersonal activity between parent(s) and child; thus, any intervention must consider the relationship between them and the nature of their interaction, whilst acknowledging the wider community and environmental influences on behaviour. Despite this evidence, as of yet, there has been no developed collaborative programmes covering both areas. As such, testing the effectiveness of such a comprehensive approach to intervention design will be crucial. Further research is needed to assess recruitment, attendance and attrition rate, acceptability of the interventions to parents and practitioners, implementation fidelity, and feasibility of evaluation measures.

Our study has a number of strengths. Our work represents a major contribution to the field of oral health development, as it is the first, to our knowledge, which has systematically developed an intervention based on sound evidence and theory. We engaged with a committed and varied group of stakeholders, including parents, commissioners, health practitioners and voluntary sector health organisations representing key disadvantaged groups. This enabled us to develop a feasible intervention which can be weaved into existing child health delivery channels. We found a number of strengths of using the IM approach, particularly, explicitly incorporating theory and evidence, and guidance on how to develop the intervention in partnership with local stakeholders. However, there are some limitations, both with the process of intervention mapping and the developed interventions which should be highlighted.

With regard to the intervention mapping process, as others have highlighted [38, 39, 44], IM is a time-consuming process and can become cumbersome when considering complex behaviours. The entire process took 4 months, with one full time researcher managing the process. The data created during the protocol can become unwieldy. For example, in the current study, we generated 6 programme outcomes, 29 performance objectives and 117 change objectives. We found it difficult to communicate this level of detail and complexity with our intervention development group. In order to deal with this challenge, we found that some element of reflexivity is required to filter and prioritise performance objectives and change objectives into a manageable number. In the current project, we did this through discussion between the research team, consultation with the intervention development group and with organisations that would be responsible for the implementation of the interventions. In addition, the emphasis on theory (identifying determinants and theoretical methods) in steps 2 and 3 of the process means that input of those with experience of behaviour change methodology is vital. Those wishing to use this approach in future should ensure that this relevant expertise is available prior to embarking on this process. Moreover, it is not possible to know at the outset what the final intervention programme will look like. This means that further research or needs assessment may be required during later stages of planning, if development takes an unexpected course. For example, early on our intervention group identified the importance of the role of parenting as a key precursor to ability to engage in PSB. This necessitates further detailed work mapping existing parenting programme provision and exploring willingness of programmes to engage with additional oral health modules. This required substantial additional resources and time. Whilst IM explicitly acknowledges the reflexivity of the process (allowing intervention development groups to move forwards and backwards along the process), it is important to be aware of challenges when adhering to planned time scales.

It is important to acknowledge that the current interventions are not without their limitations. With regard to the universal intervention, it has to be acknowledged that these services are already operating with a stretched capacity. This has been taken into account in the development of this intervention, with enhancement of these services falling largely into the provision of improved materials that can be given to parents. However, this means that not all the barriers to PSB are adequately addressed by this intervention, predominantly motivation, routine setting and behaviour regulation. In contrast, the targeted intervention does tackle all the relevant barriers but nevertheless presents challenges. The main challenge relates to the delivery settings of parenting programmes and their capability to deliver to all communities including those where parents may not speak English. A strength of the targeted PSB intervention is that parenting programmes tend to be located in deprived areas and are thus well placed to help families whose children are most at risk from caries.

Questions about reach, deliverability, uptake, success and generalisability of both the universal and targeted interventions will be fully investigated in our planned programme of research. This research will clarify the assumptions which underpin our logic model and clarify our theory of change. In summary, the intervention aims to support PSB adoption (and thus reduction in caries), by increasing motivation, and targeting key individual, interpersonal and skill-based and context-based determinants of behaviour.

## Conclusions

The current paper reports the development of a home-based parental-supervised toothbrushing intervention aiming to reduce dental caries in young children. It represents the first attempt to systematically apply evidence and theory in the development of an intervention in this context and was explicitly designed to integrate with existing delivery channels. We found intervention mapping to be useful, although not without its challenges. We recommend that groups using this methodology ensure appropriate input from an experienced multi-disciplinary group, including expertise on behaviour change theory, and that adequate time is built into timelines to allow for reflexivity in IM stages.

## Abbreviations

PSB: Parental-Supervised Toothbrushing
TDF: Theoretical Domains Framework
